# Including the gender dimension of migration is essential to avoid systematic bias in migration predictions

**DOI:** 10.1073/pnas.2500874122

**Published:** 2025-07-08

**Authors:** Athina Anastasiadou, Emilio Zagheni, Helga A. G. de Valk

**Affiliations:** ^a^Department of Digital and Computational Demography, Max Planck Institute for Demographic Research, Rostock 18057, Germany; ^b^Netherlands Interdisciplinary Demographic Institute–KNAW/University of Groningen, Den Haag 2511 CV, Netherlands

**Keywords:** migration, methods, modeling, gender, sex

## Abstract

This study examines the theoretical and methodological limitations of migration research in understanding gender-specific trends of migration. In particular, theory-driven methods suffer from the gender blindness of migration theories, while data-driven methods suffer from the scarcity of gender disaggregated migration data. This research aims to evaluate how these dual limitations affect the accuracy of commonly used migration prediction models. By analyzing migration flows disaggregated by gender, the study compares the performance of deterministic methods and probabilistic gravity-type models in predicting migrant flows with varying gender compositions. The findings reveal significant differences in the predictive performance of gravity-type models based on the gender composition of migration flows. Drawing on migration theories and case studies, the study contextualizes these findings, concluding that the lack of robust theoretical frameworks and the limited availability of gender-specific migration data have critically undermined the accuracy of current prediction and forecasting methods. The implications of this research highlight the urgent need for a critical reassessment of migration theories and methodologies through the lens of gender biases, paving the way for more inclusive and accurate migration predictions.

## Significance

Predicting international migration flows accurately is vital in order to ensure that both origin and destination countries benefit from migration. Gravity-type approaches are a class of widely adopted models used for migration foresight. However, these and related models typically neglect the gender dimension of migration. Besides their poor performance in predicting migration, we show that gravity-type models are even less helpful in predicting gender-imbalanced migration flows. This is partly because there is no theoretical framework that explains the gender composition of migration flows. We further disaggregate key dimensions of globalization of migration by gender and show that considering the levels of gender inequality at the place of origin and destination is important for improving our understanding of migration processes.

Migration is a complex process sensitive to the environment surrounding it, making it the most volatile demographic process and difficult to predict. In order to better understand and conceptualize this phenomenon, theories of international migration attempt to explain why some people migrate whereas others stay. Such theories have translated into statistical methods for migration prediction, many of which are applied by researchers and international organizations in their daily work.

According to estimates of bilateral migration flows derived from UN migrant stock data (1), female and male international migration flows from 1990 to 2020 exhibit different levels as well as different trends over time. Male migrants make up the largest group in most of the international migration corridors.^*^

A core set of theories has been influential in understanding the forces that drive migration. These theoretical frameworks have been developed across various scientific fields and mainly aim at explaining labor migration across international borders. They can be coarsely classified as Initiation frameworks including Rational-choice, Cost–benefit frameworks, Historical-Structural frameworks, and as Continuation frameworks including the Social Capital Theory (2). Initiation, Rational-choice, and Cost–benefit frameworks in migration theory comprise theoretical concepts that acknowledge migration as a process driven by, respectively, structural factors that initiate movement, individual rational decision-making, and the comparison of expected gains versus economic and social costs. Historical-Structural frameworks take into account the macrolevel circumstances that shape migration intentions and decisions by considering structural factors within potential places of origin and destination. Continuation frameworks acknowledge that the mechanisms that initiate migration are not necessarily the same as those that perpetuate migration. While these theories have, separately or collectively, contributed to explaining international labor migration, they have neglected other key drivers underlying migration motivations, like political instability, conflict, and environmental change. Gender has found its way into some of these theories as a migration motive (family migration), in line with the underlying traditional expectations of gender roles and division of labor. Gender has not been accounted for as a social divisor causing inequalities and as a form of structural discrimination intersecting with other markers of discrimination (2, 3).

Gender inequalities can enter the migration process at any stage and can be reflected in differences in migration propensities, different choices of destinations and trajectories, and different economic and integration outcomes (3, 4). Such disparities by gender^†^ raise important questions, considering that researchers overwhelmingly analyze and model migration patterns without giving proper attention to the gender dimension.

This theoretical gender-blindness is paired with a methodological one. Approaches to predict and forecast migration often apply the same assumptions to female as well as to male migrants. Many methods for forecasting net migration, or migration flows, infer the gender composition indirectly only after projecting total migration (6, 7). This is in part because the lack of comprehensive sources of international migration data, disaggregated by sex or gender, further exacerbates the challenge of developing more gender-nuanced approaches (8, 9). Migration flow data are very relevant for research as they depict in detail the direction (emigration or immigration) of the move and sometimes include demographic characteristics of the mover. Moreover, they are the best source to broaden our understanding of the “true” gender composition of immigration or emigration (5).

In this paper, we aim at assessing how the theoretical and methodological limitations described above translate into different performances of common deterministic and probabilistic prediction models by gender. Moreover, we investigate how the gender composition of migration flows varies by origin and destination country characteristics, and how the globalization of migration varies by gender. To achieve our goals, we use one of the most comprehensive datasets on bilateral international migration flows disaggregated by sex (1). We engage with migration theories and case studies to explain and contextualize our findings and conclude that the lack of sound theoretical concepts, in combination with the scarcity of gender-disaggregated migration data, has resulted in weaknesses of the methods employed in the present work to predict and forecast migration by gender. Finally, we aim to shed light on gender biases in methods and theories—to understand their structures and origins—and to suggest how they can be mitigated, and how gender theory can inform migration theory and methods.

## Results

### Gravity-Like Models Perform Poorly in Predicting Migration Flows, Even More so for Gender-Imbalanced Flows.

Gravity-like models view migration as a function of population size in the origin and destination countries, as well as the distance between them. They are widely used for predicting migration, also because their results can be interpreted rather intuitively. Extended versions of gravity models can include socioeconomic and demographic covariates for the origin and destination (10). While gravity models have been found to fare poorly for inferences and forecasts (6, 11), their potential to predict migration flows across corridors with different gender compositions has not been assessed. We use gender-disaggregated international migration flow estimates derived from migrant stock data obtained with the statistical approach pseudo-Bayes (1, 12). This dataset is the only one with global coverage that provides a bilateral migration flow matrix broken down by gender and five-year time intervals for the period from 1990 to 2020. These features are invaluable prerequisites to pursue our research aim.

Two deterministic extrapolation methods and two probabilistic methods are employed in the analysis to predict the size of the migration flows in the next time period. In order to make out-of-sample predictions to evaluate the performance of the models, we remove the observations in the last time period from the dataset and fit the models to the remaining time points. Employing the extended gravity model structure proposed by Kim and Cohen (10) yields different results for migration flows by gender. Predicting the number of women in worldwide migration flows with the OLS gravity model results in a mean absolute scaled error (MASE) that is about two to three times larger than for the predictions achieved through the simple data-driven deterministic methods (*SI Appendix*, Fig. S1). The gravity-like hurdle model produces even larger deviations from the true migration flows. The MASE for predicting the number of female migrants is by 0.4 points larger than the MASE for the gravity-like hurdle model that predicts the number of male migrants. Predicting the number of males in worldwide migration flows results in systematically lower MASE values than for predicting female flows across prediction methods. The differences are even more striking when comparing the probabilistic methods to simpler deterministic ones. This finding is consistent with the literature (6, 11) and illustrates very well how theoretical insights do not necessarily improve the model at hand. Another reason might be that gravity models can describe the spatial dimension better than the temporal one (11).

**Fig. 1.**
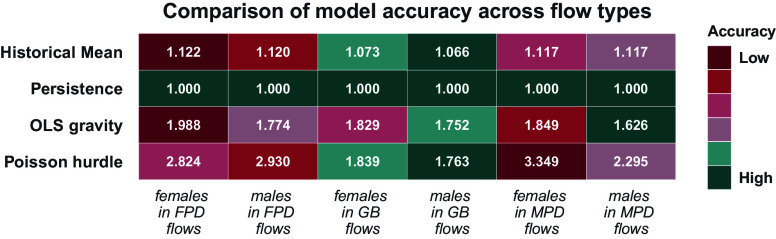
This heat map reports the mean absolute scaled error (MASE) for the comparison between the predicted values and the observed data for the number of migrants, by type of migration flow and gender, based on pseudo-Bayes estimates by Abel and Cohen (1). The flows are classified as female-predominant (FPD), gender balanced (GB), or male-predominant flows (MPD), according to the typology proposed by Donato and Gabaccia (5). For each row, representing a specific model, the accuracy is color coded, from dark red (for types of flows with low predictive accuracy, meaning high MASE values) to dark green (for types of flows with high predictive accuracy, meaning low MASE values). Across models, the predictive accuracy is generally lowest for female migrants in male-predominant flows and male migrants in female-predominant flows. For detailed descriptions of the applied methods and model specifications please see *Materials and Methods*.

In order to further examine the deviations from the true values, disaggregated by the share of females in each corridor, we employ a gender composition typology developed by Donato and Gabaccia (5). This typology identifies three categories of gender compositions in migration flows. Namely, male-predominant flows (share of females below 47%), female-predominant flows (share of females above 53%), and gender-balanced flows (share of females ranges from 47 to 53%).^‡^ Fig. 1 shows the MASE results for out-of-sample predictions of the numbers of migrants by gender in migration flows categorized based on their gender composition. We chose this metric as it has an intuitively interpretable scale and is less sensitive to outliers (13). As emerges from Fig. 1, the number of female migrants is systematically less accurately predicted across all types of flows and prediction methods compared to the number of male migrants. The MASE measures forecast accuracy by comparing a model’s mean absolute error (MAE) to that of a naive baseline (i.e. the previous period’s value), yielding a scale-independent metric that enables comparisons across time series and methods (13). Therefore, it equals one for the persistence method. In order to assess the performance of this method, we refer to the mean absolute percentage error (MAPE) values in *SI Appendix*, Fig. S2. The predicted numbers of female migrants in male-predominant flows are less accurately predicted than the numbers of males in male-predominant flows. The MASE and MAPE values for the two deterministic methods are generally much smaller, which confirms that simple deterministic methods outperform the gravity-like probabilistic ones (6, 11).

A clear pattern can be observed in Fig. 1: Across almost all methods, MASE values are the largest, in relative terms, for predicting the number of female migrants across flow types, indicating that these flows are predicted worse by almost all models. Many factors may be at play in causing the observed discrepancies. First, the assumptions applied in the models are fixed across genders and so are the covariates. However, as we will show in the remainder of this section, female and male migrants may respond differently to some gravity model components. Second, gender-disaggregated data on which data-driven approaches could be developed are scarce and oftentimes indirectly estimated themselves. Therefore, this adds another layer of uncertainty to our analysis of these estimates. Third, theory-informed approaches, like the gravity model, contain gender biases from the underlying migration theories. These biases may propagate throughout the prediction results. One potential example includes population support ratios, that might imply a different care burden, and thus obstacles to migration, by gender or other factors (we delve into this in more detail with the help of case studies in the next subsection). In other words, any difference by gender that is not captured by theory will also be ignored by the predictive models, and can result in heterogeneity in predictive accuracy, as observed in Fig. 1.

For additional error metrics, the results are similarly diverse by gender (refer to *SI Appendix*). While the MAE results indicate a worse performance for gender-balanced corridors, this can be attributed to the unscaled nature of this metric. In other words, larger flows have larger errors, in absolute terms, which leads to larger MAE values, and gender-balanced corridors are, on average, larger. The MAPE results indicate the worst performance for males in female-predominant corridors as well as females in male-predominant corridors, which is in line with the findings based on the MASE.

### Even Within One Corridor, Not a Single Theoretical Framework Can Explain the Gender Composition.

**Fig. 2.**
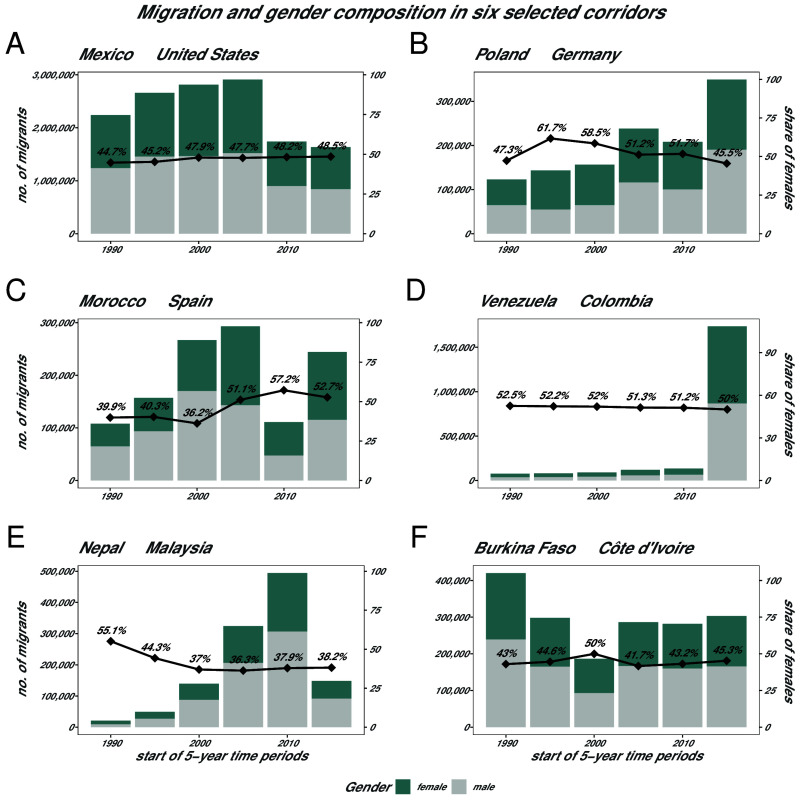
Each panel represents a different migration corridor. Panel (*A*) shows the migration corridor from Mexico to the United States, panel (*B*) from Poland to Germany, panel (*C*) from Morocco to Spain, panel (*D*) from Venezuela to Colombia, panel (*E*) from Nepal to Malaysia, and panel (*F*) from Burkina Faso to Côte d’Ivoire. The bars indicate absolute migration flows by time period in each of the six migration corridors based on pseudo-Bayes estimates by Abel and Cohen (1). Black squares indicate the share of females in the migration flow in a given corridor and time period. We can observe different developments in the share of females over time while the gender composition in absolute migration flows can differ greatly.

Every migration flow is embedded in a historical context and is a product of socioeconomic factors and political or natural events in the place of origin, as well as factors in the place of destination. These are also steered by a global system of border enforcement. Showcasing six different examples of migration corridors enables us to delve deeper into the underlying specificities that shape their gender compositions. Fig. 2 presents the share of females in different large-scale migration corridors along with the trends in the number of total immigrants. The examples were chosen to include migration corridors with a high number of immigrants as well as different trends in the share of females over time and to encompass labor, family, and humanitarian migration. While the corridors in Panel (*A* and *D*) seem to be predominantly gender-balanced over time, the trends in the number of migrants vary drastically. While Panel (*B*) exhibits an initially positive and then negative trend in the share of females over time, Panel (*C*) exhibits an overall positive one. Panel (*E*) exhibits an initially negative trend which turns into a slightly positive one over the years. Finally, Panel (*F*) is an example of a rather stable trend in the share of females over time. In order to fully understand the patterns in Fig. 2, theories and models need to account for gender norms and roles within patriarchal structures in society, and the gendered access to economic and social resources in the origin and destination countries of the migrants.

Panel (*A*) describes the corridor from Mexico to the United States, which is the largest migration corridor in the world [with 150,000 immigration events in 2022 (14)]. Its very long migration history is mainly attributed to the proximity of the two countries and the difference in their levels of economic development. For decades, since the 1940s, it was characterized by the recruitment of labor force for agriculture, and was male-predominant (15). Researchers have studied this corridor extensively and found that Mexican men are still more likely than women to migrate to the United States (16, 17). The Mexico-US corridor has seen a steady rise in female immigration over time (18). All traditional components of a gravity-like model derived from initiation frameworks like the Neoclassical Economic Theory of Migration and the New Economics of Labor Migration Theory seem to fare well in explaining this observed behavior, with the first one considering individual decision-making and the latter considering households as decision-making agents (19, 20). However, both theories neglect within-household gender hierarchies, gender-specific obstacles, and safety considerations for the journey. For the Mexico-US corridor, this observation is supported by findings from qualitative research (21). Moreover, quantitative research has found that Mexican women seem to rely more on male networks to make their migration decisions and to move to the United States, while they rely more on female networks to choose a destination and settle in it (this applies also to similar cases beyond this particular corridor) (22–25).

Panel (*B*) describes the corridor from Poland to Germany, an example where the share of female immigrants fluctuates over time. Migration from Poland to Germany used to be characterized by seasonal agriculture and domestic work. Female migrants from Poland make up almost half of the 24 h care providers in Germany’s aging society (26). Poland’s EU accession in 2004 has contributed to higher migration flows from that country to Germany but also to more seasonal migration due to the freedom of movement (27). The declining share of females could be attributed to better economic opportunities in Poland’s growing economy. In terms of transnational care chains, the population dependency ratio included in our gravity model setup might entail heterogeneous effects for female and male migrants due to the traditional gendered division of care work. The Dual Labor Market Theory views the destination’s labor markets as segmented into a primary sector for locals and a secondary sector for foreigners. It can likely explain a substantial portion of the immigration taking place in this corridor. However, women are generally more likely than men to be employed in the secondary or informal sector of any economy, often working in occupations that are regarded as low-paid (and low-status) but vital (28). In the foreign workforce there might be a clear gender hierarchy, as gender wage gaps propagate throughout all classes of the society. Findings from the literature point to the double burden of discrimination experienced by female migrants in the destination labor market (29–32). These gendered considerations are not included in the theory nor in the gravity-like models where economic opportunities are usually modeled through origin and destination countries’ GDP.

Panel (*C*) describes the corridor from Morocco to Spain which grew notably in the 1980s when labor demand in the agricultural, construction, and service sector in Spain rose. Early migrants were usually unmarried males searching for employment, together with a small number of female family migrants. The number of female labor migrants increased only in recent years due to higher demand for domestic work (and care for both younger and older persons) and due to family reunification programs by the Spanish government (33). The significant drop in immigration in the 2010–2015 period may be attributed to the financial crisis in Spain. In this corridor characterized by labor migration and subsequent family migration, family networks seem to play a more important role in facilitating female migration due to gender-specific roles and considerations (34). The Social Capital Theory of Migration (also known as Network Theory) considers the importance of social networks for facilitating migration (19). However, such differences between gendered use of certain types of networks are not considered by that theory nor by our models. Therefore, the plain number of fellow nationals in a country of destination is insufficient in predicting the number of migrants of different genders as it is currently the case in gravity-like models.

Even though sizable international migrations take place within the Global South, migration narratives focus predominantly on migration from the Global South to the Global North (35, 36). Panel (*D*) describes the migration corridor between Venezuela and Colombia, which has been characterized by humanitarian migration in recent years. Starting from the mid 2010s, Venezuela slid into a deep economic and political crisis (37). While traditionally Venezuela’s thriving oil economy attracted Colombian labor migrants and refugees of the Colombian conflict, the tides turned by 2015. Millions of Venezuelans left the country due to a total collapse of the national economy and the repressive political climate characterized by food and medicine shortages as well as a rise in violence (37). The share of females remained balanced over the years, underlining the humanitarian and forced displacement character of this exodus. Classical theories of migration generally fall short of acknowledging the plethora of motivations for migration other than labor. While few attempts have been made to conceptualize humanitarian migration [see Betts (38)], to the best of our knowledge no comprehensive theory exists that aims at explaining the gender dimension of humanitarian migration. This is despite the fact that the gendered discrimination experiences of refugees are well documented in qualitative studies (39). However, attempts to develop methods to forecast and predict flows of refugees and asylum seekers are gaining momentum (40–43).

South–South migration has historically constituted, and continues to represent, a substantial portion of global migration flows. The Nepal-Malaysia migration corridor (Panel *E*) has, over the past three decades, efficiently facilitated the flow of labor and remittances, boosting Malaysia’s economy and Nepalese households. Migrants from Nepal often migrate to find work in Malaysia’s manufacturing and service sectors (44). Thereby, it constitutes a textbook example of the Dual Labor Market Theory. Nepali migration to Malaysia has traditionally been highly male-predominant. Nevertheless, more than one third of migrants in this corridor are female [based on data by Abel and Cohen (1)]. Nepali mobility experiences are also influenced by prevailing gender norms. While younger married women often come to Malaysia to be close to their husbands, older married or widowed women also seek work abroad, sometimes to support their families or their own businesses back home (44). These findings show that there is not only a gender effect but also a gender-age effect that is currently understudied. This underscores the necessity to collect and generate age- and gender-disaggregated migration flow data for research purposes.

Migration patterns within Africa have changed over time, driven and shaped by socioeconomic, political, and environmental factors in the African context, as well as external barriers to international migration beyond the continent (35). While media narratives often portray African migration as driven by mass displacement across the largest routes, leading to the Mediterranean, most moves of Africans actually take place within the continent (35). One of the largest corridors in the region connects Burkina Faso and Côte d’Ivoire (Panel *F*). The latter is the most popular destination among migrants from Burkina Faso (45). The number of migrants in this corridor has been stagnating since 1995. Except for the period from 2000 to 2005, it has been consistently male-predominant. This is in line with the general trend that out-migration from Burkina Faso is predominantly male (45). The origins of this corridor date back to the times of French colonization, when people from densely populated Burkina Faso were forced to work on Ivorian plantations. Nowadays, most people migrate internationally in search of a job, facilitated by agreements that ease free movement, and by lower migration costs. An exception is related to interruptions during a moment of political instability following a coup d’etát and ethnic clashes in Côte d’Ivoire in 1999 (45). Recently, terrorism has disrupted Burkina Faso (46), causing internal displacement and further complicating migration patterns in the region. Gender differences in migration from Burkina Faso to Côte d’Ivoire can originate from strict gender norms and expectations. Women are often perceived as being too weak for plantation work, and prone to engage in sex work. Thus, primary migration of women has gained a negative connotation, over an extended period of time, with female migrants typically being exclusively dependent on male migrants. In the destination, these gender inequalities manifest in different access to land ownership (45). The colonial relationship indicator in some gravity models indicates whether countries share a colonial relationship as colonizer and colonized. However, colonial rule and the arbitrary partitioning of the African continent that ignored existing cultural, political, and ethnic boundaries (47) has left particular dependencies and relationships between colonized countries ignored by statistical models.

The above examples highlight the importance of understanding the special characteristics of migration corridors and how they relate to and influence the gender compositions we observe. International migration flows connect diverse systems of gender (inequality) and facilitate their interaction (48). We further analyze this concept in the next subsection.

### Higher Gender Equality at Places of Origin and Destination Is Associated with Higher Shares of Females in Migration Flows.

**Fig. 3.**
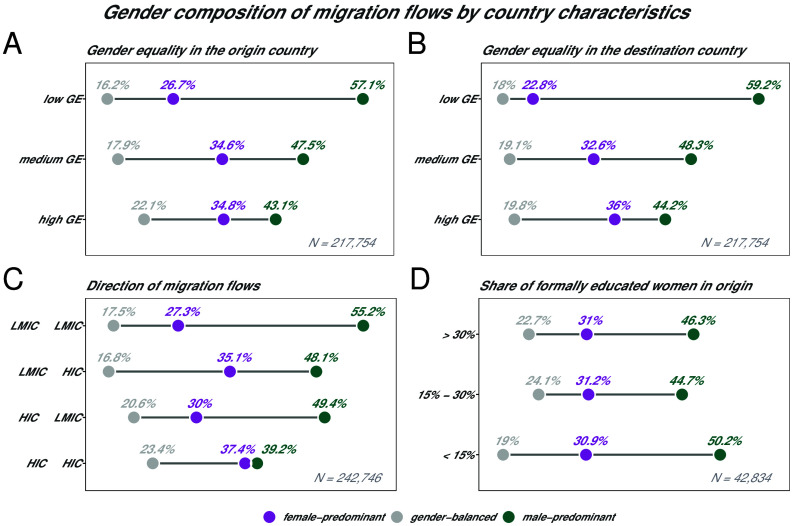
Gender composition of migration flows by different characteristics of origin and destination countries. The color-coded values indicate the percentage of flows, within each category, that are female predominant, gender balanced, or male predominant, respectively. In Panels (*A* and *B*), the three categories for gender equality (GE) are determined by the Gender Inequality Index (GII) for 2022, as provided in the United Nations Human Development Reports (52) (low GE denotes countries that appear in the *Top* third of the GII ranking, medium GE denotes countries that appear in the *Middle* third of the GII ranking, and high GE countries that appear in the *Bottom* third of the GII ranking). In Panel (*C*), the categories Low and Middle Income Countries (LMIC) and High Income Countries (HIC) are based on the World Bank definition. In Panel (*D*), the share of educated women represents the share of females in the population that obtained at least a Bachelor’s degree or equivalent. N denotes the total observations used for generating the figures. The numbers of observations vary as certain country characteristics were not available for all countries.

Gender inequalities can influence the decision and ability to migrate through gender relations and hierarchies, as well as gender status and roles in the origin society. Previous studies found that improved gender equality, and better economic rights in the origin country’s labor market, can contribute to increased emigration of women (49, 50). On the other hand, structural gender inequalities in the destination country can translate into labor market discrimination as well as other forms of discrimination (3, 39). Female migrants may face accumulated disadvantage based on labor market discrimination in the destination country due to their migrant status as well as based on their gender (30, 31, 51). In a way consistent with expectations, panels (*A* and *B*) in Fig. 3 show that higher gender equality at origins and destinations is associated with more female-predominant migration flows. More than half of the flows originating from and leading to countries with low gender equality are male-predominant. These observations suggest that different levels of gender equality in the origin and destination countries can act to retain women as well as to motivate women to migrate.

Panel (*C*) in Fig. 3 shows that the highest shares of female-predominant flows take place from Low and Middle Income Countries (LMIC) to High Income Countries (HIC), as well as from HIC to HIC. While male-predominant flows dominate all types of migration directions, they are almost equal to the share of female-predominant flows from HIC to HIC. The gender composition of some of these corridors might be partially owed to transnational care provision, a phenomenon poorly captured by classic migration theories but determining a substantial amount of female labor migration (53). Women significantly contribute to the survival of households in economies that are destabilized by transformations or by withdrawal of public welfare, in LMIC as well as in HIC (for instance, during the financial crisis in 2010) (54). At the same time, theories are biased toward explaining South to North migrations, while the majority of moves takes place within the Global South (55, 56) and gender distributions seem to differ greatly among LMIC migration flows. Therefore, focusing on data and methods that explain patterns of migration among Global South countries will contribute to our understanding of gender dynamics in international migration.

The migration and gender literature also points to educational attainment as a major driver of women’s migration intentions and moves (34, 49, 57). Having higher education can increase the desire to migrate (especially from a place with low returns to education) and improve access to resources that are necessary to facilitate migration. Panel (*D*) in Fig. 3, however, shows that female-predominant flows originate only slightly more often from countries with higher educational attainment of women.

### Key Globalization Dimensions of International Migration by Gender.

**Fig. 4.**
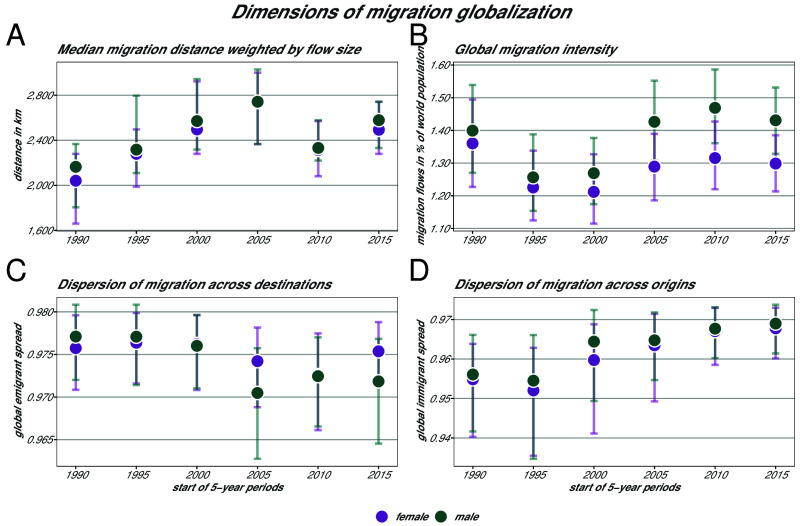
Panel (*A*) describes the median distances covered by migrants based on pseudo-Bayes estimates of flows by Abel and Cohen (1). Error bars across the panels indicate the 80% CIs obtained via bootstrap. In Panel (*B*), the *global migration intensity* is calculated as the size of migration flows as a share of world population, for the respective time period, and by gender. The *global emigrant spread* (Panel *C*) measures the level at which global migration is dispersed across destination countries, while the *global immigrant spread* (Panel *D*) measures the extent to which global migration comes from different origin countries. Both indices range from 0 to 1, with values close to 1 indicating a larger number of origins or destinations, and values close to 0 indicating a smaller number of origins and destinations chosen by migrants. For details on the methods, see *Materials and Methods*.

Czaika and de Haas (58) introduced three key dimensions in the context of understanding the globalization of migration processes: i) *migration intensity*, represented by the share of the mobile population; ii) *migration spread*, represented by the diversity of origin and destination countries of migrants; and iii) *migration distance*, represented by the average distance of migratory moves (see *Materials and Methods* for detailed calculations). All three dimensions are components of a composite migration globalization index. The process of globalization can be driven by technological and political change, for instance through increased channels of communication and interconnectedness, as well as by reduced mobility constraints and changes in labor demands and political systems (58). Empirically assessing these dimensions over time and by gender reveals the patterns that we present in Fig. 4 and that we describe in the following paragraphs.

Migration is more intense when the number of people on the move relative to the world population rises. Panel (*B*) in Fig. 4 shows how migration, in percentage of the world population, and by gender, changed from 1990 to 2020. For both genders, the rates exhibit a similar time trend but at a smaller rate for female than for male migrants. However, the differences in global migration intensity by gender are not significant. In total, only 26.1% of the corridors experienced an increase in the overall migration rate between 1990 and 2020 accompanied by an increase in the share of female migrants. This goes against the common perception of a relative increase in international migration (12, 56), but also against a common expectation of a feminization of migration (59). For the time period covered, the increase in female migration is not exceeding the one for males in relative terms.

The migration spread can be evaluated from two perspectives: the diversification of emigration patterns (namely, the growing number of migration destinations), measured by the emigrant spread, and the diversification of immigration patterns (the growing number of migration origins), measured by the immigrant spread. Panels (*C* and *D*) in Fig. 4 illustrate these two globalization dimensions for both genders. The metrics range from 0 to 1 with values close to 1 indicating a larger number of origins or destinations and values close to 0 indicating a smaller, more concentrated, number of origins and destinations chosen by migrants. The global immigrant spread exhibits a similarly positive trend over time for female and male migration, with the female immigration spread lying slightly below the male spread. This indicates a diversification of migration origins regardless of gender, and no statistically significant difference in diversification trends. On the other hand, the global emigrant spread reveals slightly, but not statistically significant, diverging trends by gender. For the female sample, it continuously declines until the 2010–2015 period and then increases. This means that female emigration was, from 1990 to 2015, directed to a declining set of destination countries and started diversifying only during the last time period. For male migration flows, the global emigrant spread follows a similar pattern until it plummets to its lowest level in the period 2005–2010 and recovers slightly the following period to drop again mildly in the period 2015–2020. This indicates that male emigration was consistently directed to almost the same or a smaller set of destination countries. This is partially in line with global trends observed by Czaika and de Haas (58) with migrant stock data from 1960 to 2000. The authors conclude that immigrant populations came from an increasingly diverse array of origins while they concentrate in an increasingly small number of destinations over time. In our data, this is in line with male migration flows but less so for female flows. These developments can have several reasons. One is that some countries have transformed from emigration to immigration countries (consider, for instance, the above examined corridor from Venezuela to Colombia), the decreasing significance of postcolonial migration patterns, decreasing emigration restrictions, and more (58).

A traditional predictor of gravity-like models for migration, among other origin and destination country factors, is geographical distance. It is also one of the three key dimensions of migration globalization. While geographical distance is often the only variable that proxies the process of the migrants’ journey in gravity-like models of migration, it might be a less relevant factor in a globalized world compared to other forms of distance, like cultural, linguistic, and legal distances (58). And its relevance might also be nuanced by gender. Entry regulations in destination countries and gender-specific migration recruitment policies are often shaped by traditional gender roles and stereotypical views of women’s societal roles, influencing how migrants move across borders (3). Migrant women were found to be less sensitive to this default determinant of a gravity model (60). Panel (*A*) in Fig. 4 shows how the median distance covered by migrants (i.e., the median distance of migration flows weighted by the size of the flows) increased between 1995 and 2010 regardless of gender. It then shrank in the 2010–2015 period and recovered afterward. The male–female difference in distance is not statistically significant.

A closer look at distance reveals that all types of flows appear over a wide range of distances and seem to be more clustered around shorter distances (*SI Appendix*, Fig. S5). However, heavily male-predominant flows are clearly taking place over shorter distances, while female-predominant flows seem to be equally distributed over the range of all possible distances. While the correlation in our sample is weak, this deserves further research as it goes against common assumptions of women as tied movers and movers over shorter distances (54, 61).

## Discussion

Viewing migration through the prism of gender inequalities aligns more closely with real-world dynamics and, as such, can enhance the predictive ability of migration theories and models. As our study shows, classic migration theories and common prediction methods overlook the gender dimension, leading to outcomes and expectations that are even less accurate than those of simple deterministic extrapolations with no theoretical underpinnings. Our analysis suggests that common methods for predicting migration flows perform differently in terms of accuracy for different migration corridors with different gender compositions. In order to investigate the potential origins of such aggregate discrepancies, we chose six selected migration corridors to illustrate gender dynamics that can explain changes in the gender composition of migration flows. By analyzing the bilateral migration flow estimates derived by Abel and Cohen (1), we conclude that many variables included in common gravity-like models for migration prediction can be associated with migration decisions differently by gender. Thereby, we explicitly challenge the theoretical and statistical foundations of gravity-type models that neglect the dimension of gender. While data-driven methods can only be as good as the data they are trained on, methods that claim to incorporate theoretical insights perform poorly in explaining patterns of diversity in the gender composition of migration flows.

In this article, we critically revisited decades-old stylized facts about gender dynamics in migration and recommend that future studies explicitly question whether the assumptions of their models hold in the same way for different types of corridors with different gender compositions. We also encourage future research to consistently analyze patterns by gender and to thoughtfully curate the variables of predictive models based on their gender sensitivity.

The narrative of the rational, cultural, and socially dominant, male migrant and the heteronormative family in migration research, a relic of the last century, should be challenged by incorporating insights from gender theory into existing explanatory frameworks for migration. This should also include developing new theories that are able to capture current patterns of globalization, emancipation, and mobility in international migration. New conceptualizations, together with enhanced gender-disaggregated migration data collection efforts and development of statistical methods for gender-disaggregation, will enable migration scholars to develop methods that perform equally well for all genders. When employing gravity-type models for their straightforward interpretation and ease of use, we encourage researchers to include gender-sensitive predictors alongside the traditional covariates. Understanding gendered migration patterns is essential for improving the accuracy of migration forecasts and for informing policy decisions in both origin and destination countries. Our findings show that traditional models systematically mispredict gendered flows, particularly in contexts with gender-imbalanced migration. For a growing number of countries, population growth heavily depends on migration. Therefore, integrating gender into predictive models becomes increasingly crucial for effective planning and policy. For instance, the differing roles of migrating mothers and fathers have distinct implications for child well-being, family structure, and care arrangements, which policymakers must consider when addressing the social impacts of migration (62, 63).

Despite their usefulness for our analysis, the bilateral flow estimates that we used in this study should be interpreted with some caution. The estimates were derived based on migrant stocks data with five-year intervals from information on previous usual residence. This means that, besides uncertainty originating from the estimation process, also measurement error from the migrant stocks propagates through the dataset. Moreover, the wide time intervals might obscure some gendered patterns in seasonal migration. Moreover, as the estimates by Abel and Cohen (1) are based on migrant stocks data provided by the UN WPP, any uncertainty induced by imputations made for countries where information on the sex characteristics of migrants are missing, will propagate through the flow estimates. Not capturing gender-differences directly but inferring them might additionally lead to a general underestimation of these differences. Potential sources of gender uncertainty in the estimation method therefore include measurement error and imputation methods applied to the stock estimates by the United Nations. While return migration could be as well a highly gendered process, this was not part of the present analysis. For exploring return migration, researchers should consider using the extended version of the dataset provided by Abel and Cohen (1) which includes information on the type of move based on information about the migrants’ place of birth. In light of this, it is important to continuously document data sources and approaches in a transparent and comprehensible manner [as done by Abel and Cohen (64)] enabling researchers to use estimates with awareness of their limitations.

With this work, we aimed to inform migration theory and estimation methodology by critically examining data and methods and by highlighting their gaps and shortcomings for predicting gendered migration flows in today’s world. We ask migration scholars and demographers to critically view the data and methods they use for their work and encourage governments and international organizations to enhance gender-disaggregated data collection efforts. For this it is inevitable to think about ways in which data are collected and how dimensions of gender can be better captured, as well critically revisit decades-old model schedules and update them with recent data. In this way, patterns of international migration by gender can be more thoroughly studied in the future. As previously expressed by Donato and Gabaccia (5, p.11), migration *“[...] statistics too are a language that can be analyzed with the methods of gender studies”*.

## Materials and Methods

For the analysis, we made use of the gender-disaggregated bilateral migration flow estimates derived by Abel and Cohen (1) based on the pseudo-Bayes method. We chose this dataset as it has maximal coverage of most countries in the world and, unlike other migration flow datasets, it provides numbers for female and male flows. The following estimation methods have been applied to a holdout dataset that was created by excluding the final time period. The models that were fitted on the pooled data are then used to make out-of-sample predictions of the number of migrants by gender and type of flow which are evaluated based on three error metrics.

### Deterministic Methods.

Two deterministic extrapolation methods are employed in the analysis of the present work to predict the size of the migration flow in the next time period. One of them is the *historical mean flow model* which describes the mean of all previous flows (6).

[1] $$\begin{array}{c} {\overset{¯}{m}}_{i,j,t}=\frac{1}{T}\sum_{t}^{T-1}m_{i,j,t}, \end{array}$$

where $m_{i,j,t}$ denotes the number of people migrating from origin country *i* to destination country *j* in time period *t*.

The second deterministic method is the *persistence model* which projects the most recent observation of a migration flow to the next period.

[2] $$\begin{array}{c} {\hat{m}}_{i,j,t}=m_{i,j,t-1} \end{array}$$

### Probabilistic Methods.

The first probabilistic method employed for the analysis is the *gravity model*. This model is popular in the social sciences to predict aggregate migration flows and is inspired from Newton’s law of gravity (10). The relationship expressed in this model is consistent with Ravenstein’s laws of migration stating that migration between two places is inversely related to the distance between them and proportional to their population sizes (65). We considered a modern version of the gravity model, that is used in state-of-the art approaches to migration flow forecasting (6):

[3] $$\begin{array}{cc} \log(m_{i,j,t}) & =\beta_{0}+\beta_{1}\log\left(D_{i,j}\right)+\beta_{2}\log\left(POP_{i,t}\right)\\ & \;+\beta_{3}\log\left(POP_{j,t}\right)+\beta_{4}\log\left(PSR_{i,t}\right)+\beta_{5}\log\left(PSR_{j,t}\right)\\ & \;+\beta_{6}\log\left(URB_{i,t}\right)+\beta_{7}\log\left(URB_{j,t}\right)+\beta_{8}\log\left(IMR_{i,t}\right)\\ & \;+\beta_{9}\log\left(IMR_{j,t}\right)+\beta_{10}\log\left(LA_{i}\right)+\beta_{11}\log\left(LA_{j}\right)\\ & \;+\beta_{12}LB_{i,j}+\beta_{13}OL_{i,j}+\beta_{14}COL_{i,j}\\ & \;+\beta_{15}\left(t-2005\right)+\beta_{16}{\left(t-2005\right)}^{2}+\epsilon_{i,j,t}. \end{array}$$

For the analysis, we included country-level data on the population of the countries of origin and destination *POP*, the share of the population residing in urban areas *URB*, the infant mortality ratio *IMR*, the potential support ratio (i.e. the number of persons aged 15 to 64 per person aged 65+ multiplied by 100) *PSR*, and the land area in squared kilometers *LA*. All population indicators (i.e. *POP* and *PSR*) have been obtained and derived from the World Population Prospects (WPP) (66). The other variables were obtained from the World Bank’s World Development Indicators at the start of each period *t* (67) with the help of the *WDI R package* (68). Indicators on shared land borders between countries *LB*, shared official language *OL*, and ever existing colonial relationship between the countries *COL* were obtained from the Centre d’Etudes Prospectives et d’Informations Internationales (CEPII) gravity model database (69). Geographic coordinates were obtained from the *countryref* dataset contained in the *Coordinate Cleaner* package in R and were used for calculating the bilateral distances to the countries’ capital cities (70).

Another probabilistic method suitable for modeling zero-inflated count data is the *Hurdle model* (6). We apply it in a gravity-like version with a binomial zero component and a Poisson count component [using the *pscl* package in R (71)],

[4] $$\begin{array}{c} m_{i,j,t}|m_{i,j,t,g}>0∼Poisson\left(\lambda_{i,j,t}\right) \end{array}$$

[5] $$\begin{array}{c} 1_{m_{i,j,t}^{c}>0}∼Binomial\left(1,\omega_{i,j,t}\right) \end{array}$$

with a covariance matrix *X* containing the same covariates as for the gravity model:

[6] $$\begin{array}{c} \log\lambda_{i,j,t}=X_{i,j,t}\beta \end{array}$$

[7] $$\begin{array}{c} logit\left(\omega_{i,j,t}\right)=X_{i,j,t}\gamma \end{array}$$

### Evaluation.

In order to assess the accuracy of the model predictions, we rely on three widely used error metrics to evaluate the out-of-sample predictions across models. The first one is the MASE usually applied in time series forecasting as it is less sensitive to outliers and its scale can be interpreted intuitively. This quantity has, on the numerator, the mean absolute error of the forecast from the model considered. On the denominator, it has the mean absolute error from a naive model where the forecasted value is equal to the quantity observed in the previous period. Values below one indicate that the model outperforms the simple persistence benchmark, whereas values above one reflect poorer performance relative to the benchmark (13):

[8] $$\begin{array}{c} MASE_{g}^{c}=\frac{\sum_{i\neq j}^{F}|m_{i,j,t=2015,g}^{c}-{\tilde{m}}_{i,j,t=2015,g}^{c}|}{\sum_{i\neq j}^{F}|m_{i,j,t=2015,g}^{c}-m_{i,j,t=2010,g}^{c}|}, \end{array}$$

where *g* denotes the migrants’ gender, *c* denotes the type of corridor (gender-balanced, male-predominant, or female-predominant), *t* denotes the start year of the time period and t=2015 refers to the period in the held out sample, while t=2010 refers to one period earlier. $\tilde{m}$ refers to the model forecast. The specifications for MAE and MASE are based on Hyndman and Koehler (13) and were adapted for our case of longitudinal data with a time series component.

The second metric used is the MAPE for point forecasts, as applied in Welch and Raftery (6):

[9] $$\begin{array}{c} MAPE_{g}^{c}=\frac{100}{F}\sum_{i\neq j}^{F}\frac{|m_{i,j,g}^{c}-{\tilde{m}}_{i,j,g}^{c}|}{m_{i,j,g}^{c}+1} \end{array}$$

with *F* denoting the total number of predicted flows, $m_{i,j,g}^{c}$ representing the true flow value, and ${\tilde{m}}_{i,j,g}^{c}$ representing the predicted flow value by gender and the three corridor types (gender-balanced, female-predominant, male-predominant). This measure has the advantage that it puts the error into context by considering the total size of the observed flow (6). While having the advantage of being scale-independent and thereby useful for comparisons across datasets, the MAPE has the disadvantage of being infinite or undefined if the true value is zero or close to zero (13).

The third error metric used is the MAE which measures the average absolute difference between the prediction of the flow and its true values (6). MAE is less sensitive to outliers compared to other common methods like rms error or mean square error (13):

[10] $$\begin{array}{c} MAE_{g}^{c}=\frac{1}{F}\sum_{i\neq j}^{F}|m_{i,j,g}^{c}-{\tilde{m}}_{i,j,g}^{c}| \end{array}$$

The MAE captures the average absolute difference between predicted and actual migration flows, expressed on the same scale as the data. The MAPE further normalizes these errors by dividing by the magnitude of the observed flow, enabling comparison of prediction accuracy across country pairs of varying flow sizes (6). The MASE measures forecast accuracy by comparing the MAE of a model to the MAE of a naive baseline forecast, making it scale-independent and suitable for comparing across time series. We prefer MASE for the main analysis as it is less sensitive to outliers (13).

### Global Migration Spread & Intensity.

The global migration intensity $MI^{\mathrm{global}}$ is measured as the rate of migration. With $worldpop_{t,g}$ representing the world population by gender and year and with *N* representing the number of all origin–destination combinations.

[11] $$\begin{array}{c} MI_{g,t}^{\mathrm{global}}=\frac{\Sigma_{i=1}^{N}m_{i,j,t,g}}{worldpop_{t,g}}*100. \end{array}$$

The global immigrant spread $IS_{g}^{\mathrm{global}}$ and emigrant spread $ES_{g}^{\mathrm{global}}$ were calculated in the following way, according to Czaika and de Haas (58), for each gender *g*:

[12] $$\begin{array}{cc} ES_{g,t}^{\mathrm{global}} & =1-\Sigma_{i=1}^{J}{\left(\frac{EM_{i,g,t}}{M_{g,t}}\right)}^{2} \end{array}$$

[13] $$\begin{array}{cc} IS_{g,t}^{\mathrm{global}} & =1-\Sigma_{i=1}^{I}{\left(\frac{IM_{i,g,t}}{M_{g,t}}\right)}^{2} \end{array}$$

with *J* denoting the number of destinations and *I* denoting the number of origins, *M* denotes the sum of all immigrants and emigrants, respectively. The number of immigrants to country *i*$IM_{i}$ and the number of emigrants from country *i*$EM_{i}$ were calculated as flows in respect to destinations and origins, respectively [as we use migration flow data instead of migrant stocks, as used by Czaika and de Haas (58)].

### Validation.

The classic gravity model has been applied to three other datasets, the QuantMig estimates for Europe (72), the estimates for Asia and the Pacific, the IMILA estimates for Latin America (73), and the Caribbean (74). For the QuantMig estimates, the number of females in male-predominant and gender-balanced corridors yield the least accurate predictions, while the number of females in female-predominant flows and the number of males in male-predominant flows is predicted closest to the true value. The MAPE values for the Asia Pacific estimates are generally much higher than for the Quantmig data. The model performs best in predicting the number of females and males in gender-balanced flows, while the worst predictions are obtained for the numbers of females and males in male-predominant flows. For the IMILA estimates, the model predicts most accurately the number of females and males in male-predominant flows. However, these estimates provide a rather mixed picture that might be owed to their rather low data quality. Detailed results can be found in *SI Appendix*, Fig. S6. The general picture obtained from the comparison across diverse datasets supports our conclusion that the accuracy of predictions by gravity-like models differ by the gender composition of different migration flows.

We performed additional robustness checks with regard to the model specification. Therefore, we considered alternative gravity model specifications, two proposed by Beyer et al. (11) and one by Cohen et al. (75). The first model we consider is a simple gravity model containing the most characteristic components: population, gross domestic product, and distance (referred to as *simple gravity* model in *SI Appendix*, Fig. S7):

[14] $$\begin{array}{cc} \log(m_{i,j,t}) & =\beta_{0}+\beta_{1}\log\left(POP_{i,t}\right)+\beta_{2}\log\left(POP_{j,t}\right)+\beta_{3}\log\left(GDP_{i,t}\right)\\ & \;+\beta_{4}\log\left(GDP_{j,t}\right)+\beta_{5}\log\left(D_{i,j}\right)+\epsilon_{i,j,t} \end{array}$$

The second model considered for the robustness check is a more complex one, also proposed by Beyer et al. (11). Besides migration regulations, we included the same variables as Beyer et al. (11) did. Data on migration regulations are unfortunately not available for each time period in our data and are hardly available for non-OECD countries. These two model specifications were chosen by Beyer et al. (11) as they differ in how they map spatial versus temporal components. The latter has been found to be predicted worse than the former by gravity-type models (11) (it is referred to as *complex gravity* model in *SI Appendix*, Fig. S7):

[15] $$\begin{array}{cc} \log(m_{i,j,t}) & =\beta_{0}+\beta_{1}\log\left(POP_{i,t}\right)+\beta_{2}\log\left(POP_{j,t}\right)\\ & \;+\beta_{3}\log\left(GDP_{i,t}\right)+\beta_{4}\log\left(GDP_{j,t}\right)+\beta_{5}\log\left(D_{i,j}\right)\\ & \;+\beta_{6}UER_{i,t}+\beta_{7}UER_{j,t}+\beta_{8}schooling_{i}\\ & \;+\beta_{9}schooling_{j}+\beta_{10}LEX_{i,t}+\beta_{11}LEX_{j,t}\\ & \;+\beta_{12}PS_{i,t}+\beta_{13}PS_{j,t}+\beta_{14}MOB_{i,t}\\ & \;+\beta_{15}MOB_{j,t}+\beta_{16}COL_{i,j}+\beta_{17}OL_{i,j}+\epsilon_{i,j,t}. \end{array}$$

The third model considered for robustness checks follows a specification by Cohen et al. (75). It is a simple geographical gravity specification. We applied it excluding the origin and destination country, and data source identifiers, which are part of the original model applied by Cohen et al. (75) (referred to as *geographical gravity* model in *SI Appendix*, Fig. S7):

[16] $$\begin{array}{cc} \log(m_{i,j,t}) & =\beta_{0}+\beta_{1}\left(t-2005\right)+\beta_{2}\log\left(LA_{i}\right)\\ & \;+\beta_{3}\log\left(LA_{j}\right)+\beta_{4}\log\left(D_{i,j}\right)+\epsilon_{i,j,t}. \end{array}$$

The population variables included in the above models, population size *POP* and mobile population *MOB* (i.e. the population aged 20 to 34 divided by the total population), were obtained and derived from the UN WPP (66). The socioeconomic variables GDP per capita (in USD 2015) *GDP*, unemployment rate *UER*, life expectancy at birth *LEX*, and a political stability indicator *PS* were obtained from the World Bank’s World Development Indicators at the start of each period *t* (67) by using the *WDI R package* (68). The distance in meters *D* between capitals of origin and destination countries was derived with the help of the *Coordinate Cleaner package* (70). The expected years of schooling, *schooling*, were obtained from the United Nations Human Development Report (76). The shared official language indicator *OL*, and the colonial relationship indicator *COL* were obtained from the CEPII gravity model database (69).

Results of the error metrics can be found in *SI Appendix*, Fig. S7 and reveal similar patterns as the results for the main model specification.

## Supplementary Material

Appendix 01 (PDF)

## Data Availability

Previously published data were used for this work (1) and processed in R (77). The scripts to download and compile the data as well as to replicate the analysis and the figures are available in a repository on GitHub https://github.com/athinaanas/gender-differences-migration-gravity (78).

## Footnotes

^*^We refer to *flows* to describe the number of people changing their country of usual residence between two time periods (based on the United Nations definition, a person is considered an international migrant if they move to a different country for a period of at least 12 mo). As the data are organized into five-year periods, the flows refer to the changes in countries of usual residence between the beginning and the end of the five-year period. With the word *corridor*, we describe a migration connection that is established between two countries over the entire study period.

^†^Gender relations, and not sex, determine the relative numbers of females and males migrating (5). Therefore, in the remainder of the article, we refer to gender differences instead of sex differences. At the same time, the dataset used for the analysis refers to the term sex. Therefore, the reader will encounter both terms in this article. We acknowledge that in current data collection systems it is rather sex than gender that is included, therefore limiting our options for analysis. Fully capturing the gender dimension would require a new approach to migration data collection and conceptualization in general.

^‡^Donato and Gabaccia (5) explicitly encourage the usage of the word “predominant” instead of “dominant” as the latter implies the possession of power over the minority group. This can lead to false conclusions as female migrants are still a more vulnerable group even if they outnumber males.
