# Supplementary material for: Including the gender dimension of migration is essential to avoid systematic bias in migration predictions
Source: Proc Natl Acad Sci U S A. 2025 Jul 8;122(28):e2500874122. doi: 10.1073/pnas.2500874122 (PMC12280947; doi:10.1073/pnas.2500874122)
Supplement: Supplementary file 1 — Appendix 01 (PDF) [file pnas.2500874122.sapp.pdf]

Supporting Information for

Including the Gender Dimension of Migration is Essential to Avoid Systematic Bias in Migration Predictions

Athina Anastasiadou, Emilio Zagheni and Helga A.G. de Valk

Athina Anastasiadou.
E-mail: anastasiadou@demogr.mpg.de

This PDF file includes:

Figs. S1 to S7
SI References

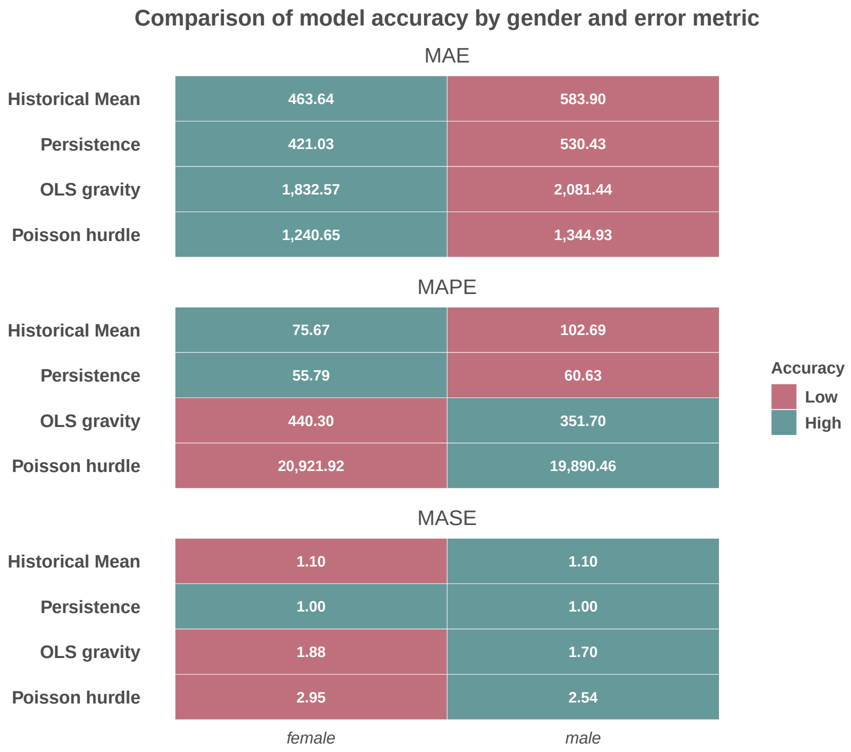

Fig. S1. This heat map reports the Mean Absolute Error (MAE), Mean Absolute Percentage Error (MAPE), and Mean Absolute Scaled Error (MASE) for the comparison between the predicted values and the observed data for the number of migrants, by gender, based on pseudo-Bayes estimates by Abel and Cohen (1). For each row, representing a specific model, the accuracy is color coded with pink representing low predictive accuracy (hence high error metrics values) and light green representing high predictive accuracy (hence low error metrics values). For the scaled metrics MAPE and MASE, the male migrants across all flows are predicted more accurately than female migrants with the exception of MAPE values for deterministic methods. For detailed descriptions of the applied methods and model specifications refer to the Materials and Methods section in the main article.

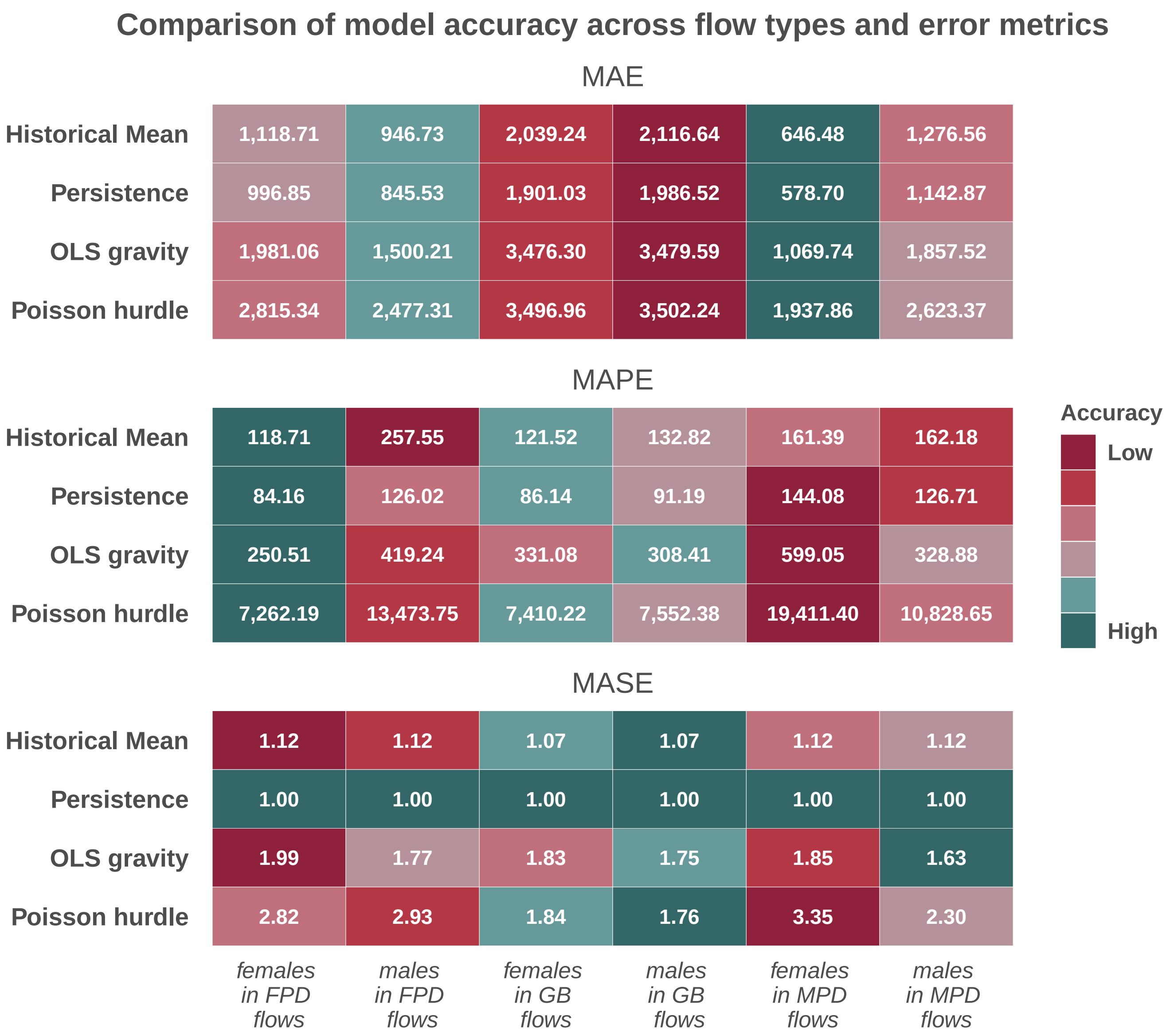

Fig. S2. This heat map reports the Mean Absolute Error (MAE), Mean Absolute Percentage Error (MAPE), and Mean Absolute Scaled Error (MASE) for the comparison between the predicted values and the observed data for the number of migrants, by type of migration flow and gender, based on pseudo-Bayes estimates by Abel and Cohen (1). The flows are classified as female-predominant (FPD), gender balanced (GB) or male-predominant flows (MPD), according to the typology proposed by Donato and Gabaccia (2). For each row, representing a specific model, the accuracy is color coded, from dark red (for types of flows with low predictive accuracy, meaning high error metrics values) to dark green (for types of flows with high predictive accuracy, meaning low error metrics values). Across error metrics, we see a high variation in predictive accuracy by type of flow. The MAE values indicate lowest predictive accuracy for gender-balanced flows. This might be attributed to the fact that this metric is not scaled by the size of the flows. For the MAPE values the predictive accuracy is lowest for female migrants in male-predominant flows and male migrants in female-predominant flows across models. The MASE values for the persistence model are 1 across all types of flows, as this metric compares the predictions with the naive forecast represented by the persistence values. For all other model specifications, it indicates lowest predictive accuracy for females in male-predominant flows as well as males in female-predominant flows. For detailed descriptions of the applied methods and model specifications refer to the Materials and Methods section in the main article.

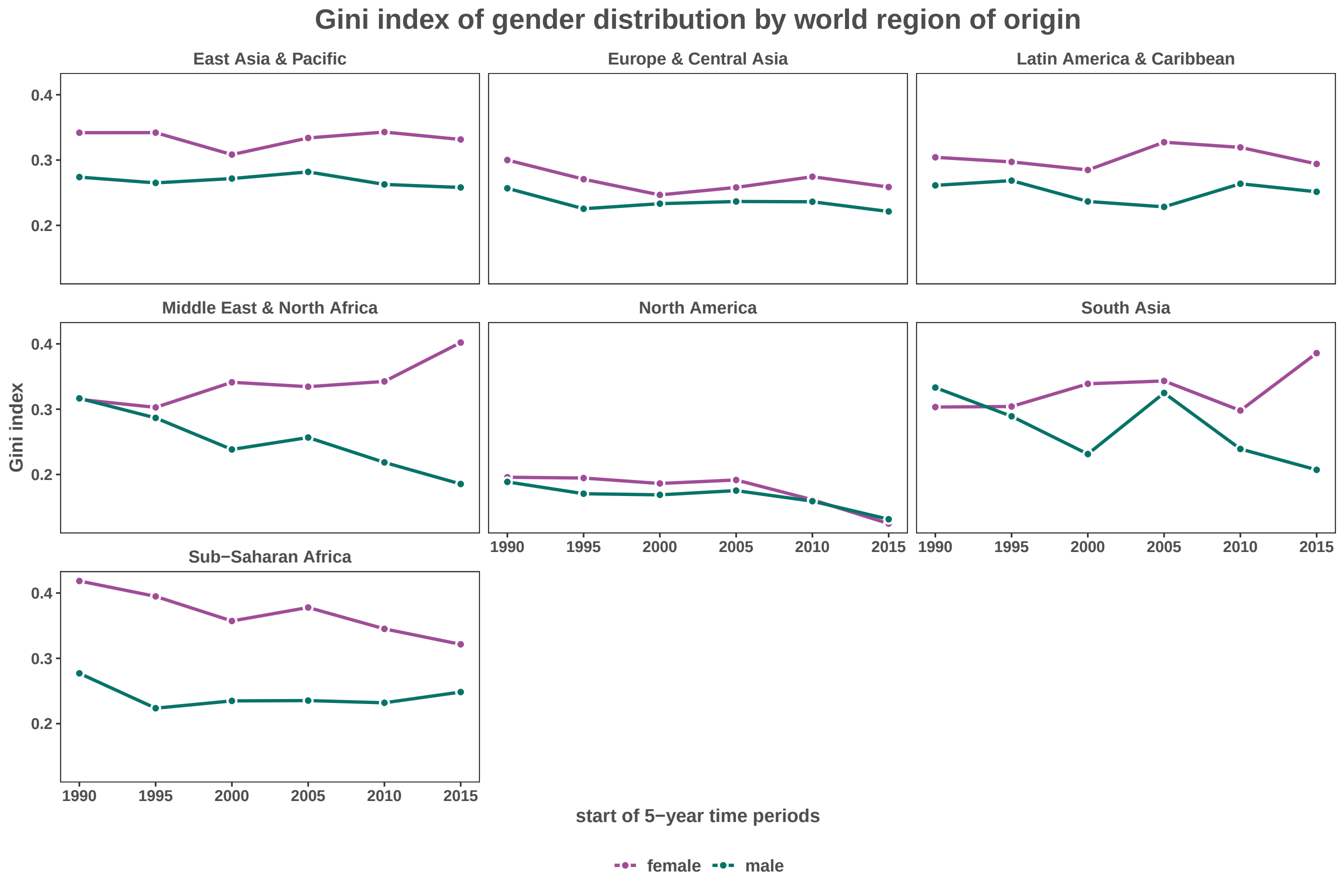

Fig. S3. Gini indices for the share of female and the share of male migrants by world region of origin over time. Values close to zero indicate an equal distribution of shares, while values close to one indicate an unequal distribution of shares. World regions were assigned based on the UN classification.

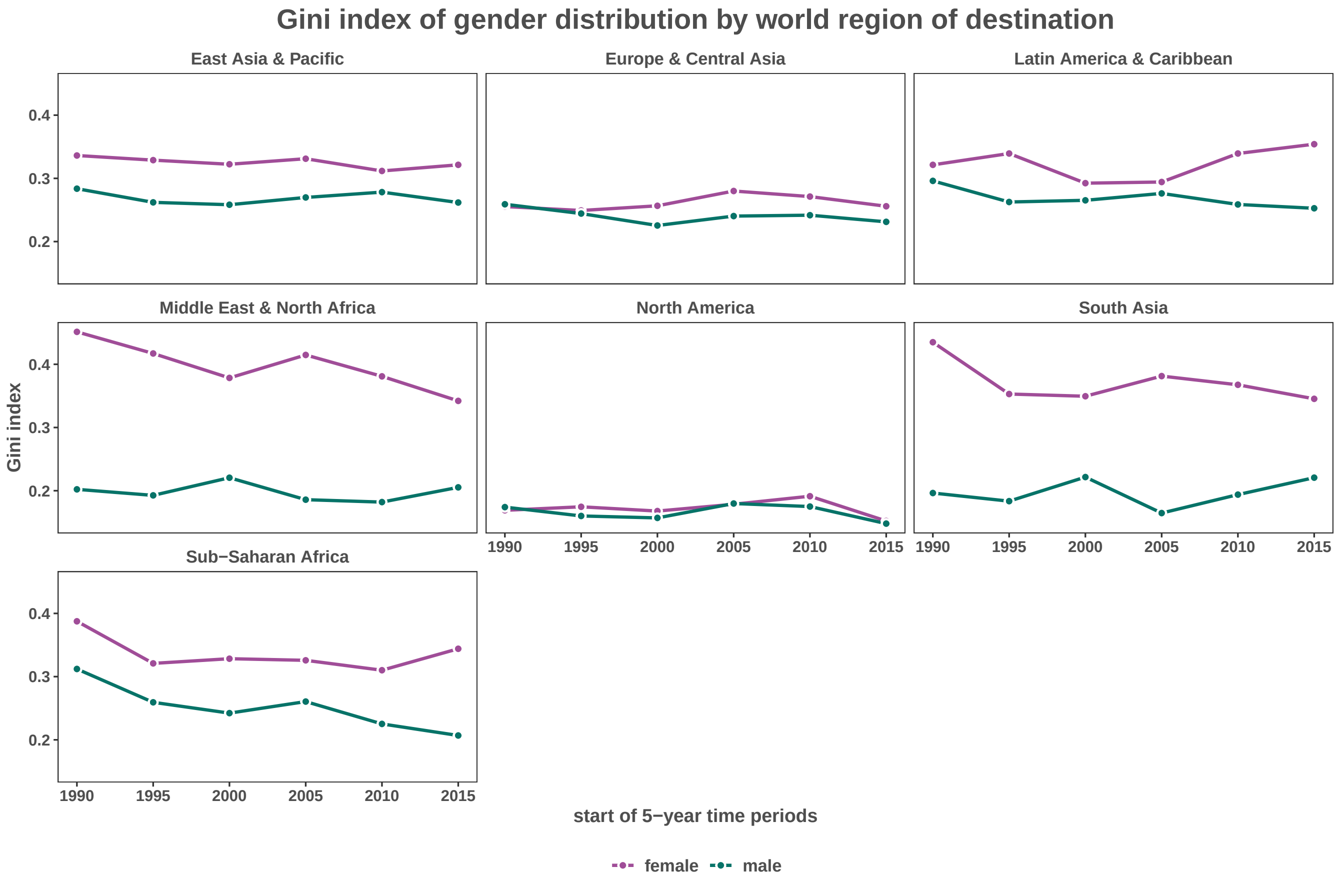

Fig. S4. Gini indices for the share of female and the share of male migrants by world region of destination over time. Values close to zero indicate an equal distribution of shares, while values close to one indicate an unequal distribution of shares. World regions were assigned based on the UN classification.

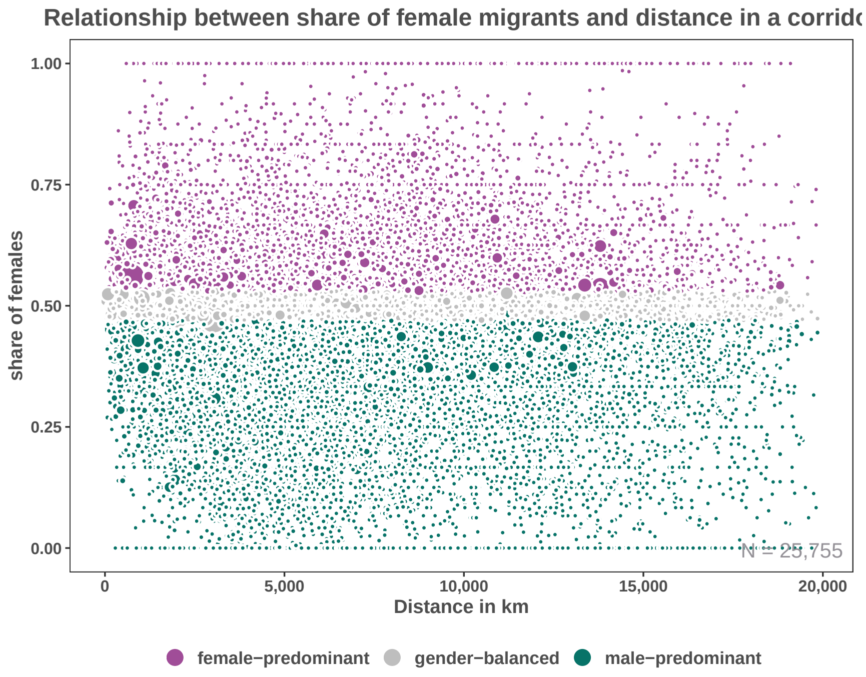

Fig. S5. This figure illustrates the relationship between distance (in km) and the share of females in all migration flows (considering only non zero flows based on data by Abel and Cohen (1)) over time. The gender composition type was assigned based on the most common type over time in each corridor. The size of the dots indicates the average total number of migrants in the respective corridor.

Comparison of model accuracy across flow types and error metrics

MAE

	females in FPD flows	males in FPD flows	females in GB flows	males in GB flows	females in MPD flows	males in MPD flows
**Global estimates**	1,981.060	1,500.210	3,476.300	3,479.590	1,069.740	1,857.520
**Europe estimates**	1,556.320	1,378.740	1,329.850	1,362.450	676.570	801.030
**Asia Pacific estimates**	3,039.250	2,804.150	5,368.470	5,461.860	2,563.290	4,008.880
**Latin America estimates**	6,166.180	4,917.510	8,849.180	8,645.790	1,344.460	2,013.010

MAPE

	females in FPD flows	males in FPD flows	females in GB flows	males in GB flows	females in MPD flows	males in MPD flows
**Global estimates**	250.510	419.240	331.080	308.410	599.050	328.880
**Europe estimates**	86.400	110.310	116.660	110.670	110.040	84.030
**Asia Pacific estimates**	142.510	168.750	546.370	494.790	566.150	409.560
**Latin America estimates**	538.020	966.730	560.780	554.810	641.920	558.100

MASE

	females in FPD flows	males in FPD flows	females in GB flows	males in GB flows	females in MPD flows	males in MPD flows
**Global estimates**	1.990	1.770	1.830	1.750	1.850	1.630
**Europe estimates**	21.830	12.710	12.350	12.430	14.270	12.360
**Asia Pacific estimates**	44.720	51.190	13.770	14.730	10.220	11.580
**Latin America estimates**			112.770	100.990	2.450	2.210

Accuracy: Low — High — NA

Fig. S6. This heat map reports the Mean Absolute Error (MAE), Mean Absolute Percentage Error (MAPE), and Mean Absolute Scaled Error (MASE) for three additional data sets of bilateral international migration flows. *Global estimates* refers to the pseudo-Bayes estimates by Abel and Cohen (1), *Europe estimates* refers to the QuantMig data set (3), *Asia and Pacific estimates* refers to the data set by Shen et al.(4), and *Latin America estimates* refers to the IMILA data (5). It compares the predicted values from the OLS gravity model specification with the observed data for the number of migrants, by type of migration flow and gender. The flows are classified as female-predominant (FPD), gender balanced (GB) or male-predominant flows (MPD), according to the typology proposed by Donato and Gabaccia (2). For each row, representing a specific data set, the accuracy is color coded, from dark red (for types of flows with low predictive accuracy, meaning high error metrics values) to dark green (for types of flows with high predictive accuracy, meaning low error metrics values). Across error metrics, we see a high variation in predictive accuracy by type of flow. Two of the MASE values for the Latin American data are not available, which is due to the different time intervals and data availability by time and gender in the data set.

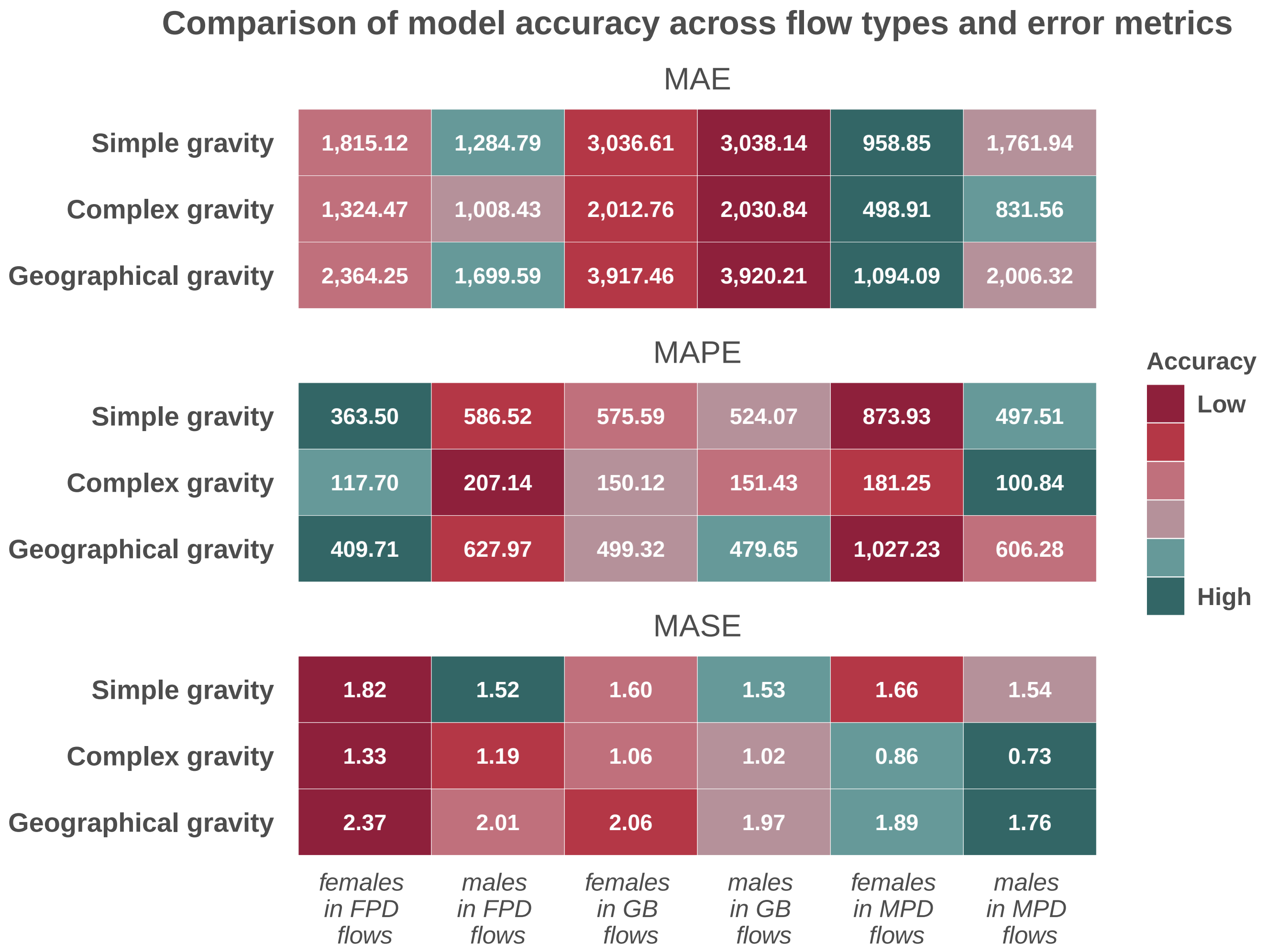

Fig. S7. This heat map reports the Mean Absolute Error (MAE), Mean Absolute Percentage Error (MAPE), and Mean Absolute Scaled Error (MASE) for three additional gravity model specifications. It compares the predicted values with the observed data for the number of migrants, by type of migration flow and gender, based on pseudo-Bayes estimates by Abel and Cohen (1). The flows are classified as female-predominant (FPD), gender balanced (GB) or male-predominant flows (MPD), according to the typology proposed by Donato and Gabaccia (2). For each row, representing a specific model, the accuracy is color coded, from dark red (for types of flows with low predictive accuracy, meaning high error metrics values) to dark green (for types of flows with high predictive accuracy, meaning low error metrics values). Across error metrics, we see a high variation in predictive accuracy by type of flow. The MAE and MAPE values mirror the results for the main model specification. The MASE values indicate worse predictive accuracy for females in all types of flows compares to males. For detailed descriptions of the applied methods and model specifications refer to the Materials and Methods section in the main article.

References

1. GJ Abel, JE Cohen, Bilateral international migration flow estimates updated and refined by sex. *Sci. Data* **9**, 1–11 (2022).
2. KM Donato, D Gabaccia, *Gender and International Migration.* (Russell Sage Foundation), (2015) Google-Books-ID: D8iZBgAAQBAJ.
3. G Aristotelous, Database: Migration estimates. *QuantMig Proj. Deliv. D6.4.* (2022).
4. T Shen, J Raymer, Q Guan, A Wiśniowski, The estimation of age and sex profiles for international migration amongst countries in the asia-pacific region. *Population, Space Place* **30**, e2716 (2024).
5. J Bengochea, E Del Fava, V Prieto, E Zagheni, Leveraging census data to study migration flows in latin america and the caribbean: An assessment of the available data sources, Technical report (2021).
